# Multiple stressors and the potential for synergistic loss of New England salt marshes

**DOI:** 10.1371/journal.pone.0183058

**Published:** 2017-08-31

**Authors:** Sinead M. Crotty, Christine Angelini, Mark D. Bertness

**Affiliations:** 1 Department of Environmental Engineering Sciences, Engineering School of Sustainable Infrastructure and Environment, University of Florida, Gainesville, FL, United States of America; 2 Department of Ecology and Evolutionary Biology, Brown University, Providence, RI, United States of America; Estacion Experimental de Zonas Aridas, SPAIN

## Abstract

Climate change and other anthropogenic stressors are converging on coastal ecosystems worldwide. Understanding how these stressors interact to affect ecosystem structure and function has immediate implications for coastal planning, however few studies quantify stressor interactions. We examined past and potential future interactions between two leading stressors on New England salt marshes: sea-level rise and marsh crab (*Sesarma reticulatum*) grazing driven low marsh die-off. Geospatial analyses reveal that crab-driven die-off has led to an order of magnitude more marsh loss than sea-level rise between 2005 and 2013. However, field transplant experimental results suggest that sea-level rise will facilitate crab expansion into higher elevation marsh platforms by inundating and gradually softening now-tough high marsh peat, exposing large areas to crab-driven die-off. Taking interactive effects of marsh softening and concomitant overgrazing into account, we estimate that even modest levels of sea-level rise will lead to levels of salt marsh habitat loss that are 3x greater than the additive effects of sea-level rise and crab-driven die-off would predict. These findings highlight the importance of multiple stressor studies in enhancing mechanistic understanding of ecosystem vulnerabilities to future stress scenarios and encourage managers to focus on ameliorating local stressors to break detrimental synergisms, reduce future ecosystem loss, and enhance ecosystem resilience to global change.

## Introduction

Multiple anthropogenic stressors increasingly affect ecological systems at the population, community, and ecosystem level [1][2]. As effects of climate change become more deleterious, a central goal of ecology and conservation biology must be to better understand, predict, and mitigate the effects of these stressors and their interactions on ecosystems [3]. When the effect of two or more stressors is the sum of their individual impacts, they interact additively. Stressor interactions can also be non-additive, where the degradation is either greater than (synergistic) or less than (antagonistic) their individual effects would predict. The potential for synergistic interactions is of particular concern as they can lead to unpredictable declines in ecological systems (i.e. ‘ecological surprises;’) [4][5]. Recent meta-analyses suggest, however, that despite initial preconceptions about synergisms as ubiquitous traits of stressor interactions, both forms of non-additive interactions may be more common than additive effects ([6][7] but see [8]). This complicates management efforts—which frequently assume that stressor interactions are additive [9]—as local interventions are predicted to produce greater than expected rewards if interactions are synergistic, but be minimally effective or potentially worsen impacts if interactions are antagonistic [10]. Without experimental field studies identifying how stressors interact, ecosystem managers will either be forced to make decisions that are decoupled from ecological understanding, or alternatively, deal with the effects of unmanaged synergies [9][11].

Coastal systems provide a powerful testing ground for investigating stressor interactions because of their exposure to a complex array of local and global, as well as acute and chronic, stressors [12][13][14]. Salt marshes, in particular, are an ideal system to study stressor interactions as they are one of the most valuable ecosystem service providers per unit area, yet are also one of the most heavily exploited and extirpated coastal ecosystems [15][16][17]. On the east coast of North America, marshes are increasingly threatened by sea-level rise (a global stressor) and marsh crab (*Sesarma reticulatum*) grazing-driven low marsh die-off (a local stressor) [14][18]. Sea level is likely to rise worldwide by a minimum of 24–55 cm (0.8–1.8 ft) over the next century due to climate change, melting polar ice caps and thermal expansion of the ocean [19], although this may be a significant underestimation [20].

Previous work has shown that as sea level increases, salt marsh cordgrass, *Spartina alterniflora*, migrates to higher marsh elevations, displacing the high marsh dominant, *Spartina patens* (hereafter marsh hay) [21][22][23][24]. This species-transition occurs because the high marsh platform experiences increased tidal inundation with sea-level rise, causing soils at this elevation to become increasingly waterlogged and, hence, more stressful for marsh hay [23]. As marsh hay dies off, its root mat that binds together the very dense, high marsh peat decomposes, thereby softening the marsh substrate, and allowing cordgrass to gradually migrate into the marsh hay zone [25]. A similar transition in plant composition and substrate hardness coincident with sea-level rise occurs at lower marsh elevations where tall-form cordgrass (typically at least 1m tall), which dominates the frequently inundated, soft substrate low marsh, gradually overtakes the stunted, short-form cordgrass (<20cm tall), which dominates the periodically inundated, harder substrate high marsh. At this transition zone, increased inundation also causes the mortality of short form cordgrass’ dense, fine root network that provides much of the rigidity to the substrate. This gradually causes cordgrass to shift belowground allocation from dense mats of fine roots to aerenchymatous rhizomes that better oxygenate the soil [25] and increase pore space, processes that work together to soften the high marsh substrate. Importantly, this loss in the structural rigidity of marsh substrates can facilitate the burrowing and bioturbating activities of marsh infauna, such as crabs [26][27]. At the same time, cordgrass at the lowest elevations experiences increased inundation and physical stress regimes, leading to marsh drowning if rates of accretion cannot keep up with the rising seas [24]. SLAMM models that account for marsh accretion conservatively estimate that a 0.30m (1 ft.) increase in sea level over the next century will cause a 13% loss in existing low marsh area due to drowning in this region [28]; however, these models do not consider how other stressors acting on marshes may interact with sea level rise and alter these predictions for marsh loss.

Concurrently, coastal predator depletion has released the herbivorous crab, *S*. *reticulatum*, from top-down control in New England salt marshes, leading to widespread consumption of low marsh cordgrass [29]. Crab overgrazing is particularly pronounced at sites with high levels of recreational fishing [18]. Tall-form cordgrass is primarily affected by *S*. *reticulatum* because the low marsh substrate is soft enough to permit this species to excavate burrows and locally graze above- and belowground cordgrass [26]. Conversely, *S*. *reticulatum* appear unable to burrow into the tough, densely rooted high marsh substrate, providing short-form cordgrass a spatial refuge from crab herbivory [30]. The interface between the low and high marsh is an abrupt substrate transition, with evidence of aboveground grazing of short-form cordgrass occurring only once crabs have completely consumed the adjacent low marsh areas (Bertness and Crotty, personal observation). However, the potential for consumer driven low marsh die-off to spread to higher elevation marsh platforms is unclear due to the constraints of the harder peat substrate. Given the degree of spatial overlap between sea-level rise and low marsh crab-driven die-off and the likely persistence of these stressors in coming decades, understanding how these stressors may interact—whether additive, synergistic, or antagonistic—will critically inform the direction and focus of regional marsh management.

Here, we use geospatial analyses and a substrate transplant experiment to quantify the historical and projected interaction between sea-level rise and low marsh die-off in New England. To test the hypothesis that high marsh cordgrass is currently protected from belowground grazing by its hard peat base, we transplanted caged and uncaged blocks of cordgrass from the high marsh to each of three experimental zones around the die-off border with a range of inundation and herbivory regimes. To test the hypothesis that sea-level rise softening of high marsh peat will increase high marsh vulnerability to grazing, burrowing, and consumer-driven die-off, we additionally transplanted naturally softened high marsh peat to experimental zones. Finally, we use conservative estimates of sea-level rise to predict future marsh softening to quantify the potential interaction between sea-level rise and low marsh die-off based on our experimental results to generate new predictions of regional marsh and ecosystem service loss.

## Methods

### Geospatial analyses

To quantify historical marsh loss at die-off and healthy marshes, aerial images of Narragansett Bay, RI (2006 and 2014) and Cape Cod, MA (2005 and 2013) were used. We delineated both total marsh area and low marsh area at 12 sites comprising the entire range of marsh die-off states (from no history of die-off to 30 years of active die-off) across the two time points. Low marsh area was delineated based on elevation, color and textural differences visible in aerial images, and field ground-truthing surveys. We quantified marsh loss, changes in low marsh area, and percent of creek banks experiencing die-off at each site. To examine the relative importance of overgrazing by marsh crabs on historical low marsh loss, we performed a linear regression between percent creek bank experiencing consumer driven die-off and percent low marsh loss over the 8-year period.

To estimate the potential expansion of the low marsh border both laterally and vertically to higher elevations with a 0.30m increase in sea level, we used LiDAR elevation data and ArcGIS software to vertically extend the current low marsh border by 1 vertical foot at each of our 12 regional sites and recalculated the low and high marsh area for each site. This analysis, in combination with recent estimates of marsh drowning accounting for accretion [28], allows us to compare estimates of marsh drowning loss (13%) with estimates of increased inundation, shifts in marsh zonation, and peat characteristics to ultimately quantify the maximum future potential area converted to low marsh and therefore vulnerable to loss if crab grazing is to keep pace with marsh softening by sea level rise.

### Substrate transplant experiment

We performed the experiment at Colt State Park Marsh in Bristol, Rhode Island (USA). Characteristic of die-off marshes in the region, this marsh is undergoing an extensive consumer driven low marsh die-off with no significant recovery [31]. First, to test whether the substrate conditions at Colt State Park are representative of those in salt marshes across the region and if patterns in substrate hardness across zones are consistent across sites, we quantified peat density using a 9kg force gauge penetrometer (Type 719; Chatillon) across three marsh elevations at 12 sites in Narragansett Bay, RI and Cape Cod, MA (*N* = 8 reps/elevation/transect; 3 transects/elevation/site). Substrate hardness was measured as the force required to push the 0.5cm diameter rod vertically into the substrate, breaching the surface level resistance, and was analyzed using a fully factorial two-way ANOVA of site and elevation.

Having verified that substrate conditions at Colt State Park are representative of substrate conditions across southern New England marshes, we excavated 36, 30x30x30-cm (LxWxH) blocks of unburrowed short-form cordgrass with straight edge shovels and moved them to the low marsh where they would be subjected to daily tidal flooding as they would be under future sea levels in October 2014. This 6 month exposure to increased inundation regimes acted to waterlog the transplant, causing mortality of fine root structures which provide much of the structural integrity of the block, and thereby decreasing substrate hardness by a factor of 2. An additional 81 blocks of short-form cordgrass monoculture were excavated from the same high marsh area in early May 2015. All replicate blocks were transplanted flush with the surface in 30x30x30-cm (LxWxD) recipient holes in their assigned treatment and elevation combinations. To test whether winter exposure to low marsh conditions decreased cordgrass biomass, 9 softened blocks were returned to the high marsh (>20m from low marsh border) in May 2015 and harvested at the end of the growing season in August 2015.

Experimental blocks were transplanted into three zones: 1) the Low Zone, 1m below the tall-/short-form cordgrass border, 2) the tall-/short-form cordgrass Border Zone, and 3) the High Zone, 1m above the tall-/short-form cordgrass border (*N* = 36 blocks per zone). In each zone, we established 9 replicates each of two ambient treatments (with and without transplant disturbance) and additionally transplanted 9 replicates of the following 4 treatments: 1) softened high marsh cordgrass (Soft), 2) hard substrate high marsh cordgrass (Hard), 3) procedural cage controls of high marsh cordgrass (CgC), and 4) consumer exclusion caged high marsh cordgrass (Cg). The consumer exclusion cages were transplanted in 30x30x50cm (LxWxD) 1cm Aquamesh cages with tops and bottoms to exclude grazing by *S*. *reticulatum*. Procedural cage controls were similar but were 2-sided with tops. The two ambient treatments (Amb) did not differ in any zone for any of the response metrics measured and were therefore pooled in the analysis.

Initial data was collected in each plot after two weeks. Substrate hardness was measured as the force required to breach the marsh surface using a 9kg force gauge penetrometer (Type 719; Chatillon). Total cordgrass tillers and crab herbivory (# tillers grazed) were scored within each plot. In a 30x30cm quadrat centered on each plot, we quantified new *S*. *reticulatum* burrows with evidence of associated crab herbivory both within and around our transplants. During the first week of August when cordgrass flowered and aboveground growth ceased, these measurements were all repeated and a centrally placed 7.5cm diameter core was harvested in each plot and returned to the lab where above- and belowground cordgrass were sorted, measured, dried and weighed. Substrate hardness and belowground biomass were analyzed with a two-way ANOVA of zone and treatment. Aboveground biomass and burrow count data were align rank transformed and analyzed with ANOVA [32]. Post hoc analyses were performed using Tukey’s HSD test with Bonferroni corrected *P* values. Grazing data mirrored biomass trends and were excluded to avoid repetitive results.

## Results

### Geospatial analyses

**Fig 1:**
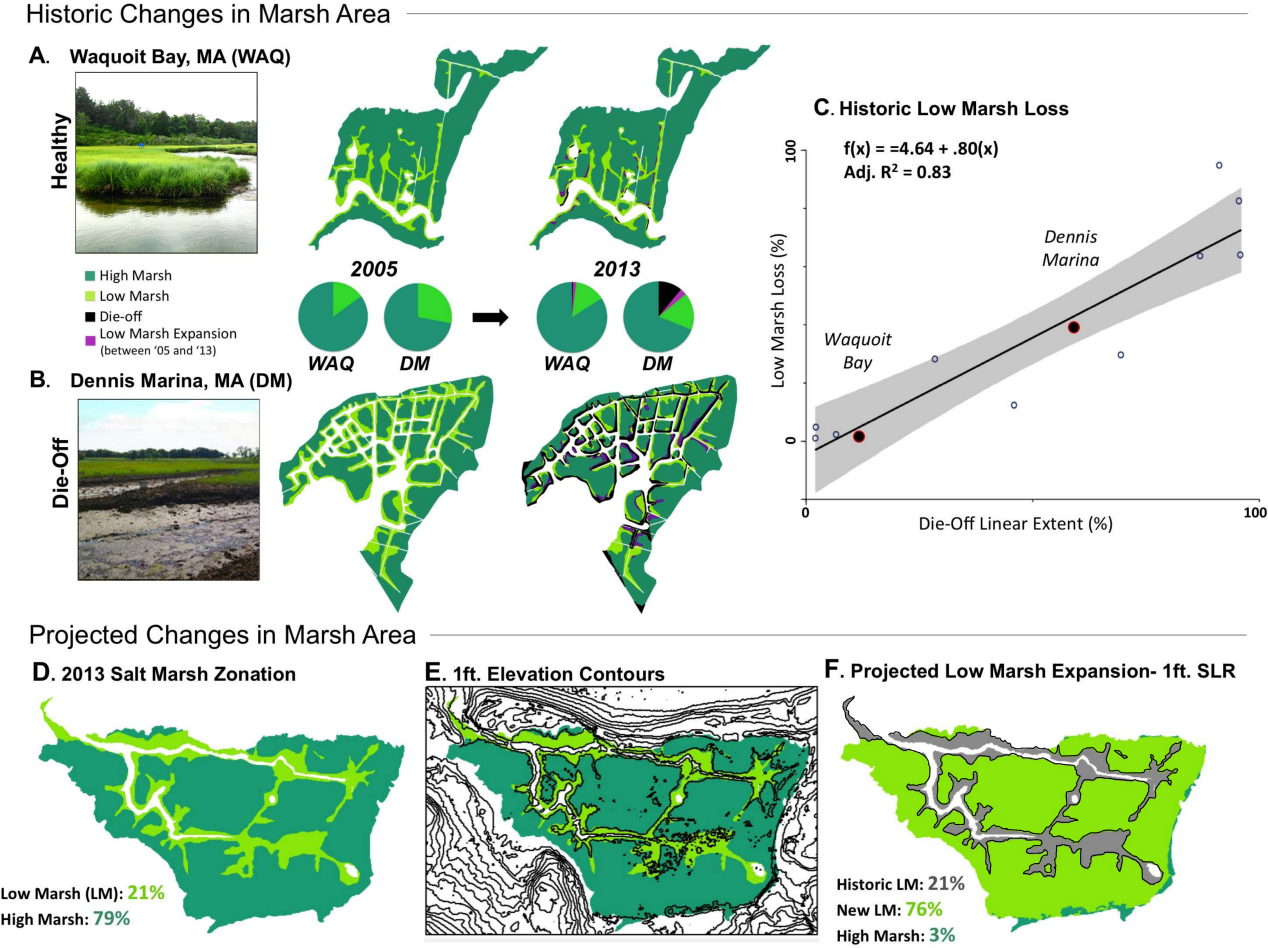
Historic and projected changes in New England marsh area. Representative healthy (A) and die-off (B) sites show different marsh loss trajectories between 2005 and 2013 and (C) linear regression analysis reveals that consumer driven die off linear extent explains 83% of the differences in historic low marsh loss across sites (*N* = 12). Representative projected areas of increased inundation, or new low marsh area, based on current low marsh border and LiDAR elevation data (D-F).

Geospatial analyses revealed that healthy salt marshes with little to no history of low marsh die-off have been largely keeping pace with sea-level rise, losing only 0.2 ± 0.1% (mean ± SEM) of their total area annually between 2005 and 2013. In contrast, marshes experiencing low marsh consumer driven die-off have lost 2.3 ± 0.6% of their total area annually, with a maximum low marsh area loss of ~95% over the 8 year period examined (62 ± 10%; die-off site mean ± SE). At the range of sites experiencing consumer-driven die-off, remaining low marsh area accounts for only 14 ± 3% of the total marsh area (Fig 1A and 1B), suggesting that soft peat availability may become a limiting factor for crab overgrazing and die-off expansion. Linear regression revealed that marsh linear extent experiencing die-off explains 83% of the variation in low marsh loss over this eight year period (Fig 1C; F_1, 10_ = 54.25, *p*<0.0001), suggesting that die-off has been responsible for far greater marsh loss in recent history than sea-level rise. However, rates of sea-level rise are predicted to increase and local SLAMM models predict 13% marsh loss over the next century due to drowning (0.30m) [28]. Using the same conservative estimate of potential sea-level rise, we quantified the total area of the marsh that may be softened, and found that due to the very gradual slope of the high marsh platform, 86 ± 3% of current marsh area will experience low marsh conditions at this projected sea level, potentially softening vast expanses of high marsh (Fig 1D–1F) if marsh vertical accretion rates cannot keep up with rates of sea-level rise.

### Substrate transplant experiment

**Fig 2:**
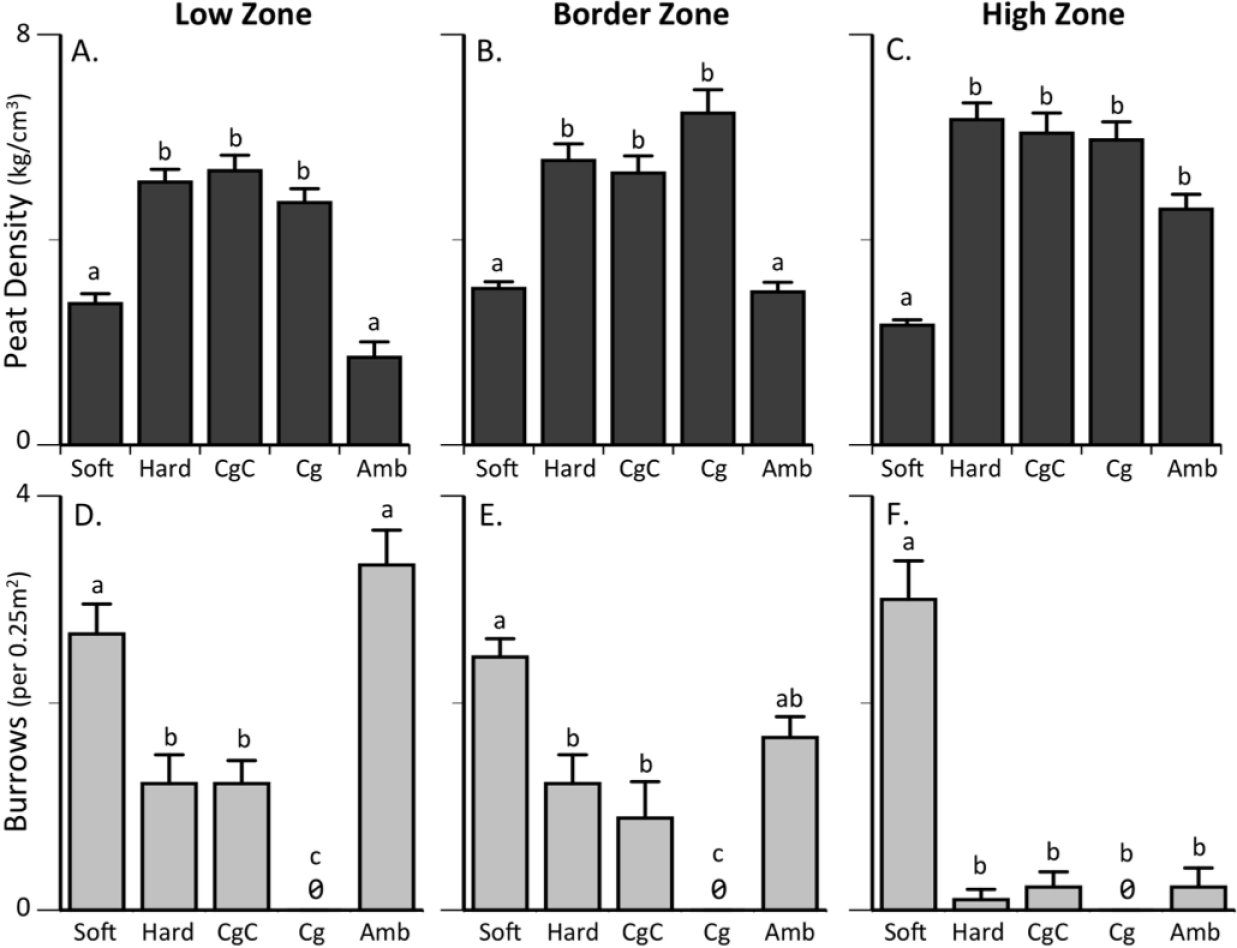
Peat density and burrow counts. Substrate hardness (kg/cm^3^) and associated burrow counts (within 50x50cm quadrat) in exposed softened peat (Soft), exposed hard peat (Hard), procedural cage control (CgC), consumer exclusion cage (Cg) and ambient (Amb) treatments in the low (A, D), border (B, E), and high zones (C, F); all means are shown + SEM. Colors highlight initial differences in substrate hardness; light gray indicates softened substrate, intermediate gray indicates hard substrate transplanted directly from high marsh, and dark gray indicates ambient substrate. Letters show significant differences across treatments as revealed by Tukey’s HSD post hoc analysis with Bonferroni corrected *P* values.

Throughout the region, mean peat density ranged from 2.6 ± 0.8 km/cm^3^ to 4.3 ± 1.2 km/cm^3^, with a representative mean substrate hardness of 3.1 ± 0.9 km/cm^3^ at Colt State Park within initial transect data (Site: F_(2,828)_ = 21.8; p<0.0001). Across all regional sites, substrate hardness was greatest in the High Zone (4.7 ± 1.1 km/cm^3^), intermediate in the Border Zone (3.4 ± 1.0 km/cm^3^), and lowest in the Low Zone (2.2 ± 1.0 km/cm^3^; Zone: F_(2,828)_ = 562.4, p<0.0001; Tukey HSD p<0.001). Following these regional trends, Colt State Park ambient substrate hardness, a proxy for peat density [33], was greatest in the High Zone (1m above cordgrass border; 4.6 ± 0.3kg/cm^2^), intermediate in the Border Zone (tall and short form cordgrass border; 3.0 ± 0.2kg/cm^2^), and lowest in the Low Zone (1m below cordgrass border: 1.7 ± 0.3kg/cm^2^; F_2, 145_ = 27.7, p<0.0001). In all experimental zones, all three hard substrate transplants (exposed hard peat: 5.7 ± 0.2kg/cm^2^, procedural cage control: 5.7 ± 0.3 kg/cm^2^, and consumer exclusion cage: 5.6 ± 0.2kg/cm^2^) had significantly higher peat density than softened treatments (2.7 ±0.3 kg/cm^2^; Fig 2A–2C; F_5, 145_ = 59.3, p<0.0001). Associated *S*. *reticulatum* burrow trends reflected these differences in peat density: in all zones, there were significantly more burrow complexes in softened treatments than in the hard substrate transplants (Fig 2D–2F; F_5, 157_ = 48.8, p<0.0001).

**Fig 3:**
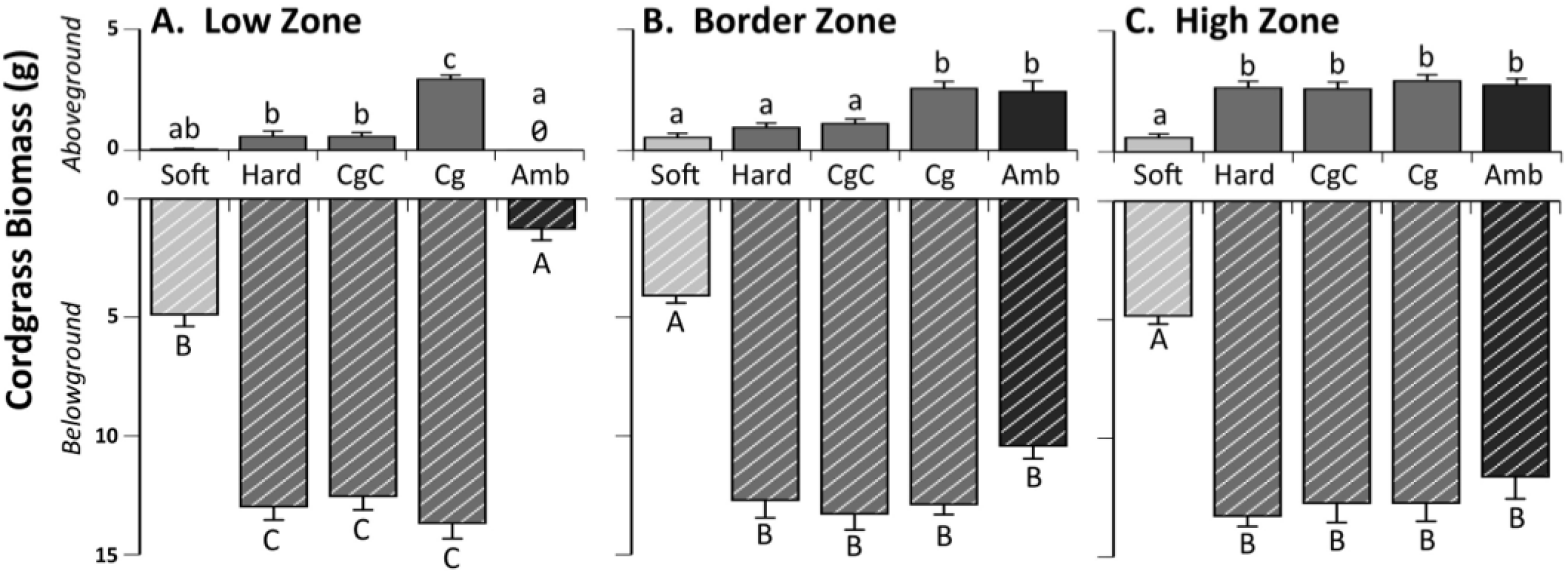
Above and belowground cordgrass biomass. Above and belowground cordgrass biomass harvested from exposed softened peat (Soft), exposed hard peat (Hard), procedural cage control (CgC), consumer exclusion cage (Cg) and ambient (Amb) treatments in the low (A), border (B), and high zones (C); all means are shown + SEM. Colors indicate initial differences in substrate hardness and letters indicate significant differences across treatments (Tukey’s HSD post hoc analysis with Bonferroni corrected *P* values).

Above (A) and belowground (B) biomass revealed significant interactions between marsh zone and treatment (A: F_8, 147_ = 17.61, p<0.0001; B: F(_8, 138_) = 19.63, p<0.0001). In the Low Zone that experiences prolonged inundation and high exposure to *S*. *reticulatum* grazing, all exposed treatments were grazed heavily aboveground, with significant biomass only remaining when consumers were excluded (Tukey HSD, p<0.001). Belowground biomass trends differed; there was no evidence of belowground grazing on any hard substrate treatment. Softened treatments, however, were similar to burrow riddled ambient plots and had significantly less belowground biomass remaining than all hard substrate treatments (Tukey HSD, p<0.001, Fig 3A).

In the Border Zone, reflecting intermediate tidal inundation and grazing exposure, all exposed treatments (softened, hard exposed, and procedural cage control) were heavily grazed aboveground. Ambient plots were composed of a mixture of tall and short form cordgrass and this is reflected in the higher remaining biomass than other exposed treatments (Tukey HSD, p<0.001). Belowground biomass was reduced by a factor of three in the softened treatments, with no evidence of any belowground grazing on any hard substrate treatments (Fig 3B).

In the High Zone, there was no evidence of any grazing on any hard substrate (consumer exclusion cage, procedural cage control, exposed hard peat) or ambient treatments (both disturbed and undisturbed) above or belowground, with no differences between treatments (Tukey HSD, p>0.20). Conversely, softened treatments were grazed heavily and had significantly less biomass remaining above and belowground than all other treatments (Tukey HSD, all p<0.001; Fig 3C). Above and belowground biomass of softened blocks that were returned to the high marsh platform (>20m from the low marsh border) did not differ from consumer exclusion cages in any zone (p>0.20), suggesting that all biomass effects within softened treatments deployed to experimental zones were not an artifact of experiencing low marsh conditions over the winter months.

## Discussion

Multiple stressors are converging on most, if not all, coastal ecosystems globally. Despite the demonstrated and anticipated frequency of cumulative impacts, our understanding of stressor interactions is inadequate [6][34][35]. Here, we provide a regionally important experimental field test elucidating the potential mechanism and scope of impact of a widespread stressor interaction. Our experimental results support our hypothesis that crab grazing is currently restricted to the low marsh. Crabs were unable to graze any hard substrate treatments belowground, indicating that die-off cannot initiate in hard substrate and, in isolation of other stressors, would be limited to existing softer, low marsh areas comprising 14% of the total area remaining at die-off sites. However, we additionally find support for the hypothesis that sea-level rise may expose high marsh peat to increased crab grazing by inundating and gradually softening the now tough high marsh peat. Our softened transplants were heavily grazed; this was especially apparent in the High Zone where crabs rapidly searched beyond the grazing front, located softened treatments, established new burrow complexes and heavily consumed cordgrass above and belowground. There was no evidence of grazing on any ambient or hard substrate transplants in this zone, since *Sesarma reticulatum* grazing is limited to a 1m area around existing burrow complexes. This supports the hypothesis that these burrowing marsh herbivores are currently stalled at the die-off border but will readily advance toward the terrestrial border as softened high marsh substrate becomes available.

Our results also identify marsh peat softening resulting from increased levels of tidal inundation as a critical factor enhancing the vulnerability of large areas of high marsh platform to consumer driven die-off. This interaction between sea level rise and crab overgrazing has the potential to precipitate extensive marsh loss because of how sea-level rise will interact with the sloping profiles of New England salt marsh systems [36]. Specifically, low marsh areas exhibit steep slopes, but cover small total areas. In these zones, as rates of sea-level rise increase, projections of marsh area loss due to drowning are relatively small. However, we show that the same incremental increases in sea level have the potential to soften large areas of the shallow sloped high marsh platform as inundation increases, and that this softened peat is extremely vulnerable to overgrazing. Therefore, while marsh drowning projections are modest, much larger expanses of area above the abrupt substrate transition between the high and low marsh may be more vulnerable to shifts in inundation and substrate hardness, depending on the ability of marshes across the region to rapidly accrete sediment and keep pace with sea level rise.

As a result of existing variation in marsh topography, substrate hardness, and future potential to vertically accrete sediment due to hydrodynamic conditions, sediment supply and variation in marsh primary production, it is likely that this inundation—softening—overgrazing sequence will occur in a patchy and temporally variable manner within and among salt marshes. Thus, our method of extending the current low marsh border one elevational foot inland to estimate the spatial extent of the low marsh under future sea level is likely a simplification of what is a fairly dynamic process. However, our experimental results suggest that at the most intense die-off sites, crabs populations are abundant but stalled at the substrate transition border, and have the potential to rapidly advance as the high marsh substrate begins to soften. In New England, our analyses estimate that 86% of current marsh area will experience low marsh inundation conditions as sea level rises by 0.30m (1ft), which has the potential to soften the high marsh peat base, and release *S*. *reticulatum* from vertical physical constraints.

**Fig 4:**
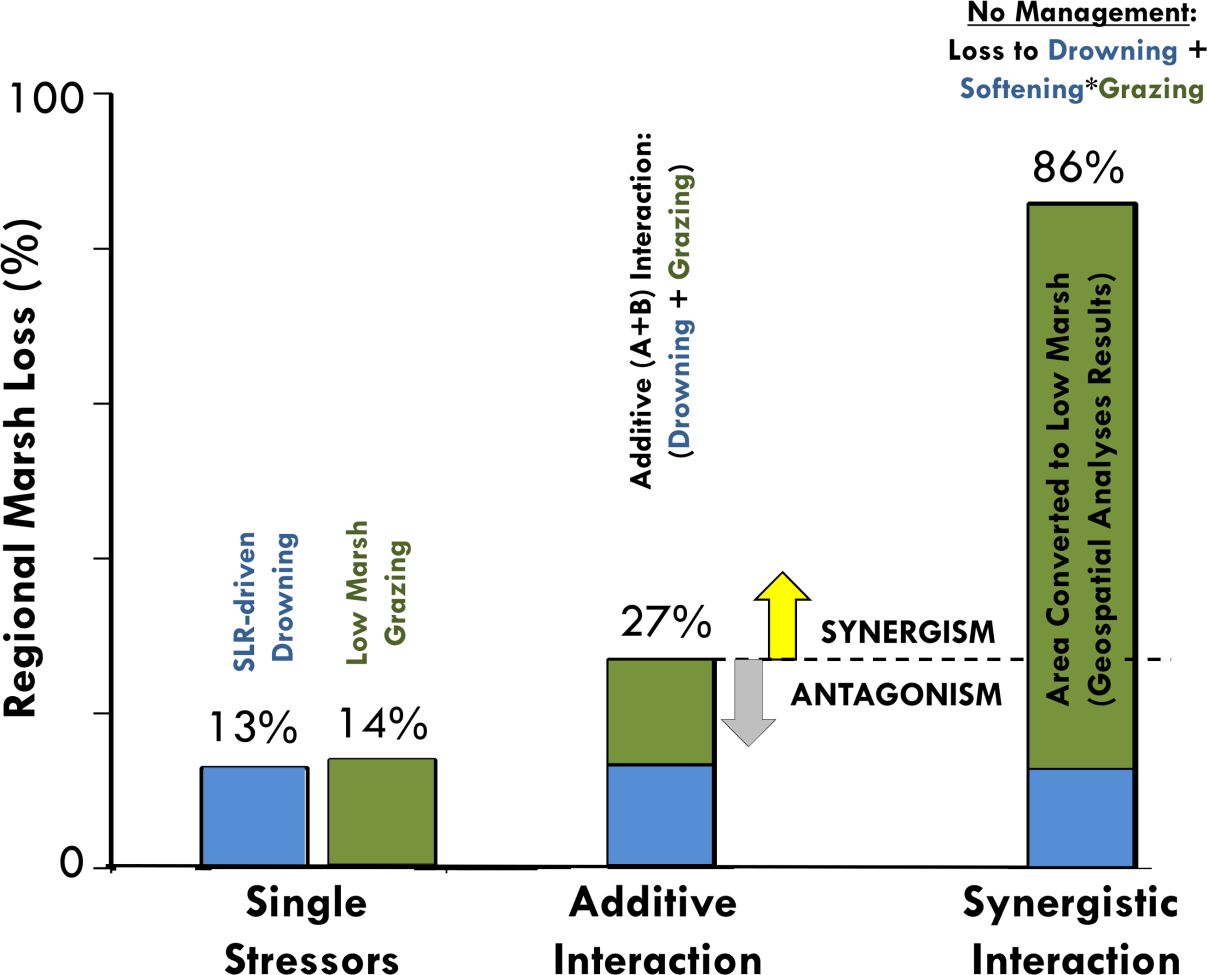
Synergistic interactions among salt marsh stressors. Marsh loss due to sea-level rise and crab driven die-off in isolation is predicted to be 13% [28] and 14%, respectively. If these global and local stressors interact additively, 27% marsh loss is projected with 0.30m increase in sea level. However, geospatial analyses reveal that up to 86% of current marsh area will be converted to low marsh with the same increase in sea level, while experimental results suggest that this new low marsh area will be softened and overgrazed by *Sesarma reticulatum*. Therefore, we find evidence that the interaction between these two marsh stressors will be synergistic, and may lead to extensive regional marsh loss without the intervention of local management.

If marsh softening regimes indeed follow elevational predictors, we anticipate that sea-level rise and crab-driven die-off will interact synergistically to drive extensive marsh loss across this region. Projections of marsh loss by sea-level rise (13%)[28] and die-off (14%; this study) in isolation pale in comparison to the area vulnerable to loss when the stressors overlap (86%; Fig 1D–1F). Together, sea-level rise and consumer driven die-off have the potential to cause three times more loss than additive effects would predict (27%), if crabs indeed exploit the entire area of salt marsh platform softened by sea level rise (Fig 4). Importantly, these potential long term marsh loss scenarios are decreased by a factor of six (86% to 13%) at sites where healthy predator populations are maintained, controlling grazing by *S*. *reticulatum*, where the primary cause of marsh area loss is drowning by rising seas. Research that examines where sediments eroded by *S*. *reticulatum* burrowing activities become redistributed—to the surface of the high marsh platform, slump into tidal creeks, or are exported from the system—as well as work elucidating the ability of salt marshes in the region to accrete sediment and adapt to increases in sea level is needed to further refine these predictions.

Crab outbreaks and intensive overgrazing of salt marsh foundation species are not simply a regional phenomena and have been extensively reported in both South America [37] and China [38]. Indeed, outbreaks of bioturbating organisms and consumers that have destabilizing effects on structure and function of systems are seen across a vast array of ecological communities, including but not limited to coral reefs, rocky intertidal zones, seagrass beds, mussel beds, as well as sandy and rocky shores [39][40][41][42][43]. Therefore, many coastal ecosystems may be similarly vulnerable to the interactive affects of global human impacts, such as sea-level rise, and local population dynamics of bioturbating organisms and/or dominant grazers.

Globally, coastal systems are extremely vulnerable to climate change driven stressors, especially sea-level rise [14][44]. Local stressors commonly overlap with these global stressors, disrupting the biogenic habitat-forming organisms that build and maintain many coastal systems and increasing vulnerability to drowning and other global stressors [2][6][45][46][47]. A mechanistic understanding of stressor interactions will enable coastal managers to evaluate whether their action to curb local stressors are likely to promote rapid recovery and increase ecosystem resilience [48]. While there is little that local management can do to curtail global stressors like sea-level rise [10], our results reveal that the benefits gained from ameliorating local stressors can be significant. Furthermore, over the next century, climate change and overfishing are expected to be dominant drivers of future trends at all levels of community organization in coastal and marine systems [49][50] but are rarely studied in conjunction [48]. In New England marshes, management that reduces localized overfishing of marsh predators has the potential to reduce projected marsh loss by a factor of six over the next century. Regional managers therefore have the ability to maintain ecosystem functioning in the short term to ‘buy time’ for larger scale solutions to be implemented. Conversely, if predator populations remain depleted and die-off initiates at all sites in southern New England, large expanses of marsh may be vulnerable to overgrazing and marsh loss. In coastal storm protection and carbon sequestration services alone, this area of enhanced vulnerability and loss is worth $161.5 million/year to southern New England [16][51][52]. Ultimately, the most cost-effective management strategy will be to dismantle synergies by focusing on stressors that initiate and act locally to break the dramatic loss that occurs when they overlap with multiple global stressors.
